# Clinical Outcomes Evaluation of Combined Valgus and Chiari Osteotomy Inconsistent with Patient Satisfaction

**DOI:** 10.1155/2018/2409656

**Published:** 2018-04-18

**Authors:** Akira Hozumi, Kennichi Kidera, Ko Chiba, Takayuki Shida, Makoto Osaki

**Affiliations:** Department of Orthopaedic Surgery, Nagasaki University Graduate School of Biomedical Sciences, Nagasaki, Japan

## Abstract

The Japanese Orthopaedic Association Hip-Disease Evaluation Questionnaire, which is tailored to Japanese lifestyles, has recently been developed in Japan as a patient-reported outcome measure. In this study, combined valgus and Chiari osteotomy were evaluated using the JHEQ and JOA scores. The subjects were 42 hips of 39 patients with a mean age at surgery of 45.3 years. The mean follow-up period was 95.3 months. Radiological osteoarthritis stage, preoperative and postoperative JOA scores, JHEQ score at final follow-up, and patient dissatisfaction with hip joint status rated on a visual analog scale were evaluated. The factors that affected patient dissatisfaction were also identified using multiple regression analysis. Radiological osteoarthritis stage at final follow-up was either maintained or improved in 85.7%. The mean JOA score improved from 57.2 preoperatively to 78.7 at final follow-up. The JHEQ score at final follow-up, however, was low, at 43.3 points. Patients who were comparatively satisfied accounted for 47.6%. Of the JHEQ subscales, movement had the lowest scores, and this was the subscale that had the greatest effect on patient dissatisfaction. The present results suggest that the results of JOA score are inconsistent for postoperative patients' satisfaction after CVCO, and patient-based evaluation tool must also be used.

## 1. Introduction

In Japan, secondary osteoarthritis of the hip caused by developmental dysplasia of the hip (DDH) is the leading cause of osteoarthritis of the hip [1, 2]. For the patients with primary osteoarthritis of the hip, clinical improvement is difficult to achieve even by remodeling and reconstructing the bone and hip morphology. On the other hand, improvements of the joint cartilage in DDH can be induced in many cases by the construction of biomechanical elements by osteotomy. Osteotomy has therefore been proactively used to treat comparatively young patients, even if arthritic changes are advanced. Chiari osteotomy is a surgical procedure that reduces pain by improving coverage of the acetabulum and promoting reproduction of the fibrocartilage derived from the joint capsule [2–4]. Valgus osteotomy of the hip [5–10] is a widely performed procedure to achieve joint stability, repair, and regeneration using the proliferative changes of osteoarthritis. Since 2002, we have been performing combined valgus and Chiari osteotomy (CVCO) to treat patients with advanced or terminal-stage osteoarthritis of the hip whenever possible.

Relatively good postoperative outcomes have been reported after hip osteotomy. However, they were mostly assessed by the perspective of healthcare providers. Therefore, they are not always sufficient for the more detailed evaluation of patients' physical symptoms, social hardships, and advantages as a result of surgery.

In recent years, evaluation criteria such as quality of life (QOL) and satisfaction from the patients' standpoint have been regarded as important [11–15].

The Japanese Orthopaedic Association Hip-Disease Evaluation Questionnaire (JHEQ) has recently been developed in Japan as a tool for evaluating patients' quality of life. It uses criteria specific to hip joint disease and is tailored to Japanese lifestyles. It comprises an assessment of patient dissatisfaction with hip joint status as scored on a visual analog scale (VAS) together with 20 questions, with three subscales (pain, movement, and mental status) with a possible score of 28 points each, for a total possible score of 84 points. The JHEQ is a patient-based evaluation tool, with each question scored by the patient on a five-point scale, and its reliability and validity have been established by Matsumoto et al. [16, 17].

In this study, the clinical outcome of CVCO was evaluated using the Japanese Orthopaedic Association hip score (JOA score) and the JHEQ, and the results were compared. Furthermore, the association of each subscale with the VAS score was also investigated. These results were then used to identify factors that affected patient dissatisfaction with this operation.

## 2. Subjects and Methods

The study subjects were 42 hips of 39 patients among the 73 patients who underwent CVCO at our hospital between January 2002 and July 2011. The JHEQ survey was self-administered and distributed either by post or at the end of a hospital visit. The survey was sent by post only to patients who were able to undergo an examination within six months before or after it was posted.

The subjects were 4 men and 35 women, with a mean age at surgery of 45.3 years (range 22–57 years); the mean follow-up period was 95.3 months (range 35–153 months). Preoperative stage was categorized according to the radiographic system defined in 2010 by the Investigation Group into Coxarthrosis and Acetabular Dysplasia in Japan [18, 19] as preosteoarthritis (2 hips), initial stage of osteoarthritis (4 hips), advanced stage of osteoarthritis (14 hips), and the terminal stage of osteoarthritis (22 hips).

The preoperative roundness index of the femoral head [20] was 59.6%.

The items investigated were patient dissatisfaction with hip status at final follow-up (VAS), correlations between the VAS score and the JOA and JHEQ subscale scores, and correlations with other factors such as osteoarthritis stage, and the factors that had the greatest effect on the VAS score were investigated.

Statistical analysis was carried out using Spearman's rank correlation coefficient and multiple regression analysis, with *p* < 0.05 regarded as significant.

This study was approved by the institutional review board (approval number: 17091101), and the requirement for informed consent was waived owing to the retrospective nature of the study.

## 3. Results

**Figure 1:**
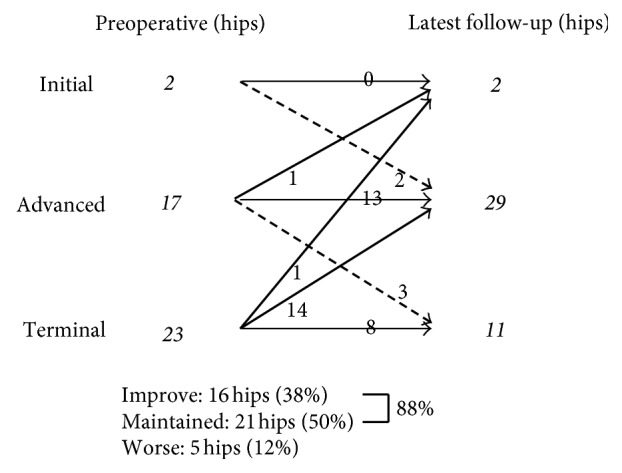
Changes in osteoarthritis stage. At final follow-up, osteoarthritis stage is either maintained or improved in 88.1%.

In terms of radiographic analysis at final follow-up, arthritis stage was either maintained or improved in 88% of cases, with advanced seen in only 5 hips (Figure 1).

**Figure 2:**
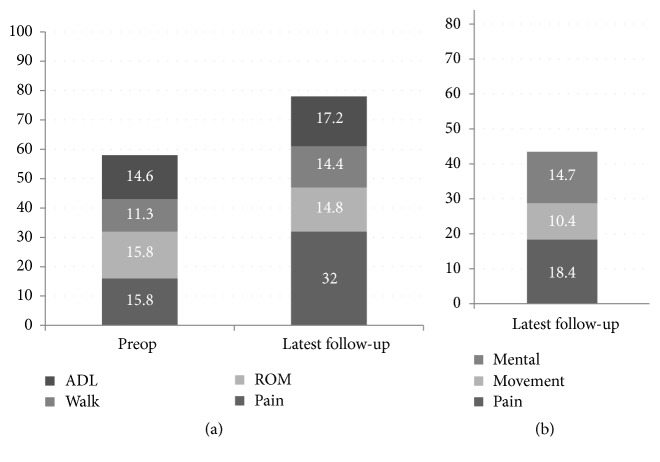
Change in JOA score and JHEQ score VAS score at final follow-up.

The mean JOA score improved from 57.2 points preoperatively to 78.7 points at final follow-up (Figure 2(a)), but the total JHEQ score at final follow-up was lower, at 43.3 points (scoring rate 49.3%) (Figure 2(b)), lower than the JOA score. Movement subscale had the lowest scores among the various factors included in the JHEQ.

**Figure 3:**
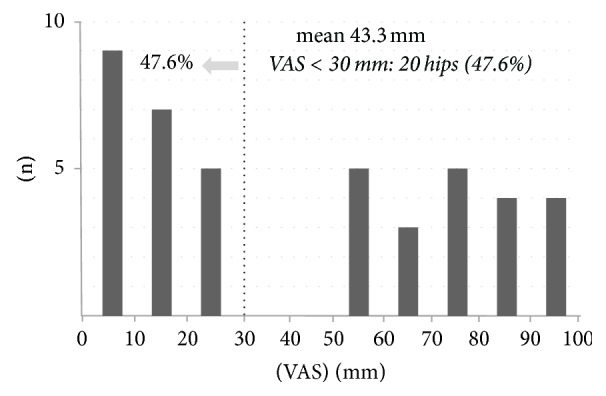
VAS score at final follow-up. The mean VAS score is 43.3 mm, and 20 hips (47.6%) are comparatively satisfied, with a VAS score ≤ 30 mm.

The mean VAS score at final follow-up was 43.3 mm. Twenty patients (47.6%), were comparatively satisfied, with a VAS score ≤ 30 mm (Figure 3).

There was some correlation with each factor between the JHEQ and JOA scores, but they did not have the strong correlation (≥0.7) (Table 1).

In the correlation of VAS and each subscale of JHEQ and JOA, movement, mental status subscale, and total scores of JHEQ had strong correlations (≥0.8) (Table 1).

According to the correlations between the VAS score and the roundness index of the femoral head, preoperative osteoarthritis stage, osteoarthritis stage at final follow-up, age at surgery, follow-up period, and the preoperative JOA subscale score, only the osteoarthritis stage at final follow-up had a weak correlation (Table 2). Based on these correlation results, multiple regression analysis with JHEQ subscales as explanatory variables was performed to investigate their effects on the VAS score. The movement subscale had the greatest effect on VAS score (Table 3). Specifically, the mean scores for 6 of the 7 items that make up the movement subscale were <2 points, indicating some sort of difficulty (Table 4). For questions in the mental status subscale, the scores which influenced daily activity tended to be lower because of hip disease (data not shown).

## 4. Discussion

Recent reports have pointed out the importance of including subjective assessments from the patient's perspective in postoperative evaluation, in addition to assessment by healthcare providers, and the existence of discrepancies between these two methods of evaluation has been demonstrated. McGee et al. [14] found that discrepancies between objective and subjective assessments tend to emerge when patients who had undergone total hip arthroplasty had systemic complications. Liberrmann et al. [15] reported that discrepancies tended to be more common in patients with low levels of postoperative satisfaction.

In this study, assessment by the JHEQ clearly indicated a poorer outcome than when the JOA score was used, and although there was some degree of correlation between subscales, these correlations were not strong. This result was similar to those of studies reported by other authors.

In particular, the present findings suggested that the results of the JHEQ better reflect factors including the following: (1) JOA and JHEQ scores differed with respect to movement-related items that required deep hip flexion; (2) the effect on mental status of the gap between excessive expectations of surgery and the reality; and (3) the effect on the movement subscale of the negative effect on the other joints by a persistent or increased leg length discrepancy.

Although there have been few reports of postosteotomy patient-based assessments, Kaneuji et al. [21] looked at long-term outcomes after rotational acetabular osteotomy (RAO). They reported that the mean JHEQ score at final follow-up was 57 points, and mean patient dissatisfaction as measured by the VAS score was 22.8 mm, which were better outcomes than in the present study. This may have been due to the different preoperative factors prevalent in CVCO compared with RAO. CVCO is often carried out on patients whose preoperative stage is comparatively advanced, and although the surgery does provide some degree of pain relief, patients still have poor hip function and low levels of postoperative activity compared with those who undergo RAO. Furthermore, it cannot provide leg length adjustment, meaning that limping may persist or even worsen over time. This may affect the other joints, which in turn affects the movement subscale. It also reflects the fact that, although this operation is time-consuming and physically invasive, it is also time-saving surgery, and factors such as the psychological anxiety and stress entailed in having to undergo a second operation. These factors may have an effect on mental status. Kaneuji et al. [21] carried out an analysis by preoperative osteoarthritis stage, and they found that those who underwent RAO for advanced osteoarthritis had significantly lower scores only on the movement subscale, a similar tendency to that observed in the present study.

Actual subjective comments received from patients included a comparatively large proportion of movement-related complaints that affected activity levels, such as back pain from limping or difference in leg length, and increased pain in the joints of the opposite leg following surgery.

These findings suggest that the JOA score alone is insufficient for postoperative evaluation of CVCO, and another patient-based evaluation tool such as the JHEQ must also be used. Due to recent improvements in the durability of materials and advances in surgical procedures for total hip arthroplasty, CVCO is now being used to treat younger patients, and the number of patients for whom it is indicated is tending to decrease. Further studies are required to clarify the indications for this procedure.

A limitation of this study is that the JHEQ score is difficult to use for preoperative and continuous evaluation.

## 5. Conclusions

The JOA and JHEQ were used to evaluate clinical outcomes of CVCO, and they were compared as methods of postoperative assessment. The movement and mental status subscales of the JHEQ were strongly correlated with the VAS score.

The present results suggest that an objective evaluation tool from the healthcare providers' perspective is inadequate for postoperative assessment after CVCO, and a patient-based evaluation tool must also be used.

## Figures and Tables

**Table 1:** Correlations between JHEQ and JOA subscales.

JHEQ	JOA				Total
	Pain	ROM	Gait	ADL	
Pain	0.655	0.509	0.596	0.564	-
Movement	0.538	0.662	0.648	0.581	-
Mental	0.626	0.509	0.655	0.534	-

Total	-	-	-	-	0.838

The value (modulus) is Spearman's rank correlation coefficient.

**Table 2:** Correlations between VAS scores and individual factors.

Factor	Correlation coefficient (*r*)^*∗*^
Roundness index of the femoral head	0.057
Stage of osteoarthritis (preop)	0.040
Stage of osteoarthritis (latest follow-up)	0.515
Age	0.014
Leg length discrepancy (latest follow-up)	0.349
Duration of follow-up	0.006
JOA score (preoperative)	
Pain	0.102
ROM	0.007
Walk	0.077
ADL	0.107

^*∗*^The value (modulus) is Spearman's rank correlation coefficient.

**Table 3:** Factors affecting the VAS score (multiple regression analysis).

	Regression coefficient	Standard error	*p* value	95% CI
Constant term	96.2	6.56	<0.01	82.74–109.3
Pain	−0.22	0.60	0.71	−1.44–0.99
Movement	−2.21	0.74	0.004	−3.71–−0.71
Mental	−1.77	0.57	0.003	−2.94–−0.61

**Table 4:** JHEQ items (movement).

Question (movement)	Points^*∗*^
It is difficult for me to climb up and down stairs	1.64
It is difficult for me to get up from the floor and tatami	1.59
It is difficult for me to squat	1.54
It is difficult for me to use a Japanese-style toilet	1.02
It is difficult to get in and out of a bathtub	2.00
It is difficult to cut my toenails	1.14
It is difficult to put on my socks	1.54

^*∗*^Patients' answers—strongly agree, agree, uncertain, disagree, and strongly disagree—are scored as 0, 1, 2, 3, and 4 points, respectively.
